# *Batrachochytrium dendrobatidis* strain affects transcriptomic response in liver but not skin in latitudinal populations of the common toad (*Bufo bufo*)

**DOI:** 10.1038/s41598-024-52975-8

**Published:** 2024-01-30

**Authors:** Niki Chondrelli, Emily Kuehn, Sara Meurling, Maria Cortázar-Chinarro, Anssi Laurila, Jacob Höglund

**Affiliations:** 1 Animal Ecology/Department of Ecology and Genetics, Uppsala University, Uppsala, Sweden; 2 MEMEG/Department of Biology, Faculty of Science, Lund University, Lund, Sweden

**Keywords:** Ecology, Evolution, Immunology

## Abstract

*Batrachochytrium dendrobatidis* (*Bd*) is a fungal pathogen that has decimated amphibian populations worldwide for several decades. We examined the changes in gene expression in response to *Bd* infection in two populations of the common toad, *Bufo bufo*, in a laboratory experiment. We collected *B. bufo* eggs in southern and northern Sweden, and infected the laboratory-raised metamorphs with two strains of the global panzoonotic lineage *Bd*-GPL. Differential expression analysis showed significant differences between infected and control individuals in both liver and skin. The skin samples showed no discernible differences in gene expression between the two strains used, while liver samples were differentiated by strain, with one of the strains eliciting no immune response from infected toads. Immune system genes were overexpressed in skin samples from surviving infected individuals, while in liver samples the pattern was more diffuse. Splitting samples by population revealed a stronger immune response in northern individuals. Differences in transcriptional regulation between populations are particularly relevant to study in Swedish amphibians, which may have experienced varying exposure to *Bd*. Earlier exposure to this pathogen and subsequent adaptation or selection pressure may contribute to the survival of some populations over others, while standing genetic diversity in different populations may also affect the infection outcome.

## Introduction

The spread of infectious diseases presents a grave threat to biodiversity. Habitat loss, pollution, human-wildlife interaction, wildlife trade, and invasive species act in concert to transmit pathogens and aggravate their effects^1,2^. Amphibians are among the taxonomic groups most sensitive to these disturbances and have been experiencing rapid population declines over the last several decades^3,4^. The chytrid fungus *Batrachochytrium dendrobatidis* (*Bd*), causing the disease chytridiomycosis, has been implicated as a major agent in this global extinction event^3,5,6^. The ancestral lineage of *Bd* is endemic to East Asia and has, through the trading of amphibians as pets, medicine and food, spread across the globe and diversified^7^. The global panzootic lineage of *Bd* (*Bd-*GPL) is responsible for many of the amphibian declines recorded in the last decades^8^.

Amphibians affected by chytridiomycosis experience an inability to regulate electrolytes as the disease attacks the keratinized layers of their skin, preventing the transfer of ions to and from the environment. This leads to disruption of the electrical signals within the animal’s body, particularly with regard to the heart, and many individuals with chytridiomycosis ultimately die of cardiac failure^9,10^.

*Bd* is implicated in the declines of at least 501 amphibian species worldwide, 90 of which are (presumed) extinct in the wild^6^. While the fungus has been reported to infect about half of the amphibian species tested (54% of 1854; Ref.^11^), the severity of chytridiomycosis appears to vary widely between species and populations. Even within species, chytridiomycosis can be inconsistent in its severity^12,13^. Thus, amphibians and *Bd* are being studied worldwide to assess what factors influence disease susceptibility and how the pathogenicity of *Bd* varies on an interspecific basis and between populations.

The outcome of chytridiomycosis can be influenced to a significant extent by the host's immune responses^14^.There is evidence of amphibian species recovering from the disease due to a shift in their immune response, in the absence of a shift in *Bd* pathogenicity^15,16^. For example, Voyles et al.^17^ reported a more effective *Bd* inhibition from skin microbiota of *Bd*-exposed compared to *Bd*-naïve *Atelopus varius*, a frog native in central America, where catastrophic declines due to *Bd* were observed in the 2000s. Other mechanisms of resistance and tolerance to *Bd* may include innate immune responses, such as the secretion of skin antimicrobial peptides in response to *Bd* infection^18^, or adaptive responses, for example upregulation of B and T lymphocytes in skin tissue of *Bd*-infected amphibians^19^.

Variation in the Major Histocompatibility Complex (MHC) class IA and IIB has also been shown to affect levels of susceptibility to *Bd* in amphibian species^20–22^. Directional selection for certain MHC alleles has been observed in populations of both frogs and toads following exposure to *Bd*^21–23^. At the transcriptomic level, regulation of the MHC as well as other immune-related genes appears to vary between susceptible and resistant individuals. Interestingly, MHC-related and, more broadly, immune genes are upregulated in susceptible infected populations, while little change is observed in resistant populations^13,24,25^.

Despite the evidence of immune system activation in many amphibians after exposure to *Bd*, immune response is not always effective in combating infection, and a growing body of literature suggests that in some species *Bd* may have immunosuppressing mechanisms^26–28^. Rosenblum et al.^28^ showed a decrease in the expression of some immune genes in *Bd*-infected *Silurana tropicalis*, while Ellison et al.^26^ demonstrated a reduction in lymphocyte gene expression in spleen from infected *Atelopus zeteki* compared to control individuals. Further examples include a potential interference of *Bd* with skin antimicrobial peptide levels in *Litoria serrata* through peptide degradation or inhibition of their production^29^, as well as a failure to activate an adaptive immune response in *Silurana tropicalis*^30^.

It is also observed that *Bd* strain can influence the infection outcome, and different isolates can have dissimilar effects on the same host. The majority of studies on the topic focus on the effects of different strains on the survival of either tadpoles^8,31,32^ or adult frogs^33,34^. Eskew et al.^35^ extend their observations to include the effects of different strains to the host gene expression in the skin of two frog species. The susceptible species, *Rana sylvatica*, suffered high mortality when infected with the more virulent of the two *Bd* strains used, despite activating an adaptive immune response, while the resistant species, *Rana catesbeiana*, had similar gene expression and high survival when infected with either *Bd* strain.

In this study, we use lab-raised common toads, *Bufo bufo*, from southern and northern Sweden. Importantly, these populations differ in their genetic makeup. After the last glacial maximum, Sweden was colonized by two different lineages of *B. bufo*, with the southern populations harboring a much larger genetic diversity^36^. The lower genetic diversity of the northern populations is reflected in the reduced diversity of MHC class IIB haplotypes in the north as compared to the south of Sweden^22^. The two populations also differ in developmental strategies, with northern populations reaching metamorphosis at a smaller mass^37^. Meurling et al.^37^ challenged newly metamorphosed common toads with two different strains of *Bd-*GPL, a 2008 UK isolate (UKMal 01) from a wild alpine newt (*Icthyosaura alpestris*) and a 2015 Swedish isolate (SWED-40-5) from a wild green toad (*Bufotes viridis*, see O’ Hanlon et al.^7^ for information on the isolates). They demonstrated that survival was size-mediated, with smaller individuals (primarily the ones from the North) suffering higher mortality and the UK strain causing higher mortality overall.

The present study aims to elucidate the mechanisms behind the population and treatment (*Bd* strain) differences observed in the previous infection experiment^37^, by examining the transcriptional responses of the metamorphs’ skin and liver to infection by *Bd*. As the skin is the primary site of fungal infection, the transcriptional consequences of *Bd* infection in the skin are of great interest. The liver is involved in a variety of metabolic processes, which are affected by the availability of electrolytes and other components transported across the skin. The inclusion of the liver provides us with insight as to how the disruptions occurring at skin-level affect other physiological processes. Additionally, the liver is a site of macrophage production, making it a relevant organ for analysis in the context of disease pathophysiology^38^. Based on the survival results of Meurling et al.^37^, we hypothesized to see a stronger immune response in southern individuals as well as in those infected with the Swedish strain, as those two groups exhibited higher survival during the infection experiment.

## Results

### Individuals included in the RNAseq

We sequenced RNA samples from 106 individuals infected with a Swedish or UK *Bd* strain or sham-infected with *Bd* medium. All animal samples were collected from a previous experiment by our group^37^. The samples consisted of paired liver and skin samples from 75 individuals, plus 11 unique liver and 20 unique skin samples (N = 181 samples, Table 1, Supplementary Table S1). Some samples were not paired, as we excluded low RNA quality samples (RIN < 7.5). After filtering out two skin samples with low read counts after sequencing (< 500,000 reads), as well as one liver sample clustering with the skin samples in the PCA (presumably a mislabeled sample, Supplementary Fig. S1) we ended up with 85 liver and 93 skin samples. No control individuals from either population died (Table 1) while over half of all infected toads died before the end of the 30-day experiment. The majority of deaths occurred in the toads from the northern population (N = 33 dead individuals from the North, N = 5 from the South).

**Table 1:** Sample sizes and mortality information for *Bufo bufo* metamorphs from which tissue samples were used in the RNAseq experiment (106 individuals).

Treatment	Population	Individuals (survivors)	Skin samples (from survivors)	Liver samples (from survivors)
Control	North	13 (13)	11 (11)	13 (13)
	South	19 (19)	16 (16)	16 (16)
Infected SWE	North	20 (8)	18 (7)	14 (6)
	South	16 (12)	14 (10)	13 (11)
Infected UK	North	24 (3)	22 (3)	18 (0)
	South	14 (13)	13 (12)	11 (11)

In parentheses in column 3, number of individuals surviving to the end of the infection experiment. In columns 4–5, number of samples for each tissue from surviving individuals.

### Differential gene expression between tissues (liver vs skin) and survival status (surviving vs dead individuals)

Gene expression differed greatly between the two tissue types; 75% (N = 16,627) of all (N = 22,181) genes in the analysis are either up or down regulated in skin compared to liver; *full model* in Supplementary Table S2, Supplementary Figs S2 and S3). Within each tissue, expression differed depending on survival status, i.e. there were many differentially expressed genes when comparing individuals that survived until the end of the experiment (hereafter “surviving”) and those that did not (hereafter “dead”) (Supplementary Figs. S4 and S5, models *skin full, skin infection status, liver full, liver infection status* in Supplementary Table S2). Functional analysis revealed that skin samples from dead individuals were functionally enriched for genes related to the regulation of cell death, the expression of glucocorticoids, and stress and some immune functions (Supplementary Table S3), while they were depleted for structural genes as well as collagen-related cellular components (Supplementary Table S4). For the liver samples, fewer differentially expressed genes were discovered using the same threshold (9952 vs 3849 genes, *skin full* vs *liver full* in Supplementary Table S2). In dead individuals, overexpressed genes were enriched for developmental terms (Supplementary Table S5), while no terms were enriched for the surviving individuals.

For all further analysis, we kept only the samples from individuals surviving at the end of the experiment and split the dataset by tissue, as liver and skin samples had such dissimilar expression profiles.

### Differential gene expression in skin samples from surviving individuals

**Figure 1:**
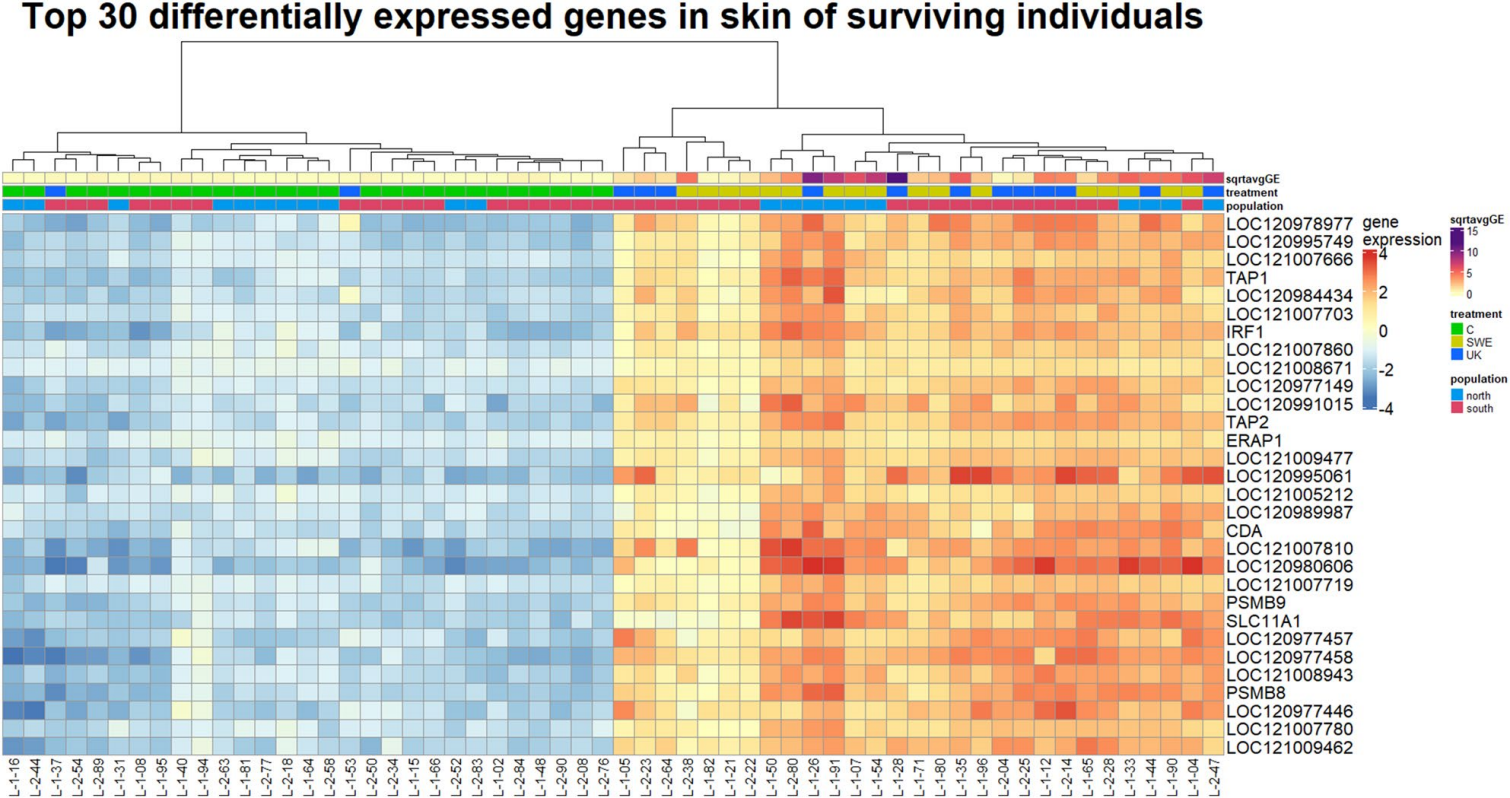
Heatmap of top 30 differentially expressed genes in skin samples of surviving individuals. Gene expression is normalized counts centered around the mean expression for each gene. The dendrogram is a hierarchical clustering based on sample similarity. sqrtavgGE: the square root of the average genomic equivalents of Bd (see Meurling et al.^37^); the minimum value (0) indicates individuals with no detectable Bd load, treatment: *C* = control, SWE and UK Bd strain, population: north or south. Heatmap generated with the R package ComplexHeatmap, version 2.14.0.

**Figure 2:**
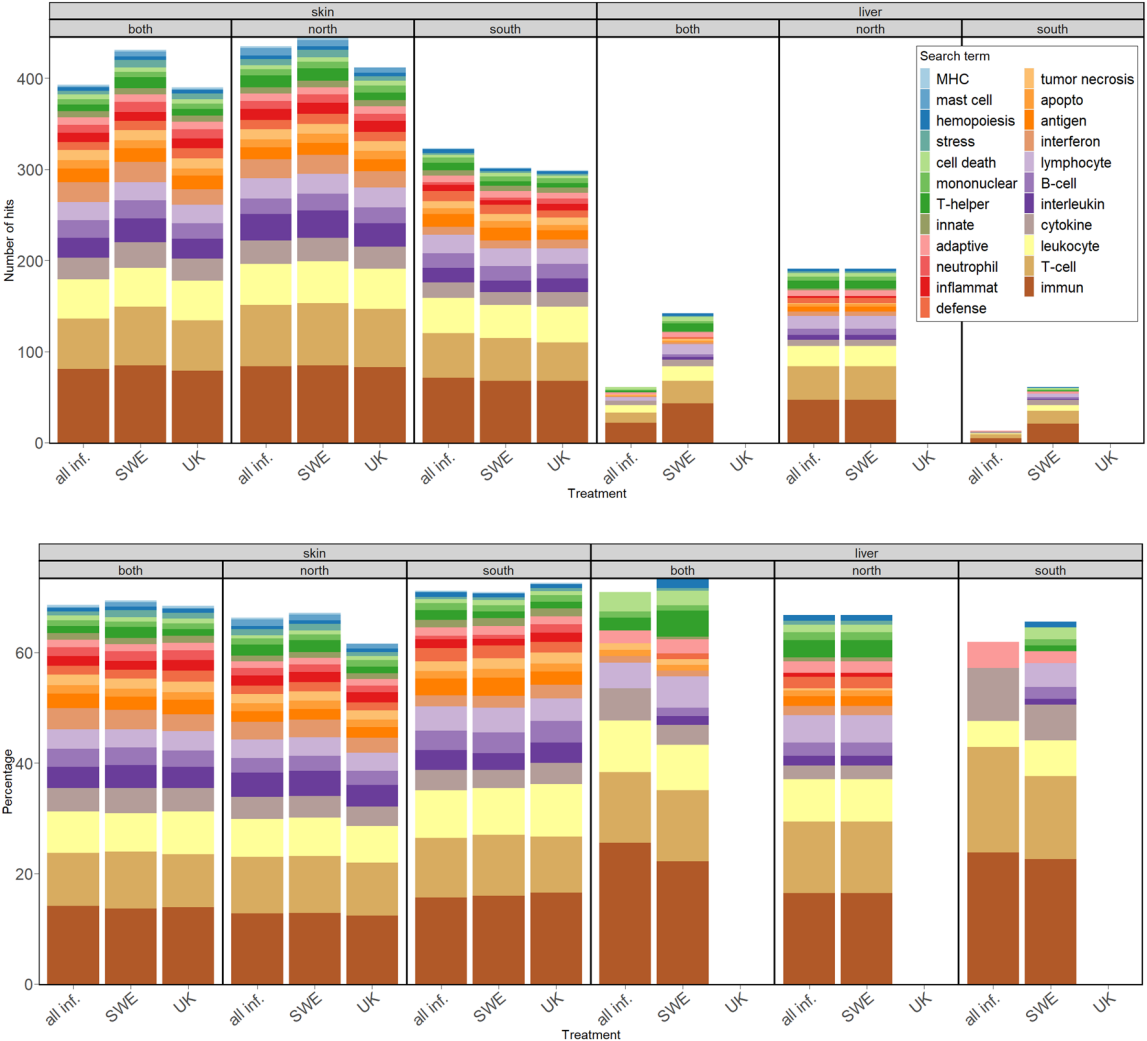
Number (2a) and percentage (2b) of immune related search terms in comparisons between infected and control surviving individuals using the Homo sapiens database in g:Profiler. The data is split by tissue (skin–liver), population (north, south or both) and treatment (all inf.: all infected individuals, SWE: individuals infected with the Swedish Bd strain, UK: individuals infected with the UK Bd strain. All comparisons are with control individuals of the same category. Percentage (2b) refers to the percentage of immune related terms over all the gene ontology terms related to biological processes and enriched in each comparison). Only 11 Gene Ontology (GO) terms were functionally enriched in liver samples from individuals infected with the UK Bd strain, none of which were related to immune terms. No liver samples from surviving UK-Bd infected individuals from the north were sequenced, so there are no GO terms associated with those samples. This also means that the GO terms for all infected individuals compared to controls and SWE-Bd infected individuals compared to controls are identical in liver samples from northern toads. Finally, liver samples from southern individuals infected with the UK Bd strain were not enriched for any of the GO search terms.

The skin samples of surviving individuals were primarily differentiated by infection status (8805, or 42% of 20,863 genes differentially expressed), and secondarily by population (7217 or 7337, 34 – 35% of all genes, depending on model used: *skin alive* or *skin alive—inf status* in Supplementary Table S2). There were few genes differentially expressed between the two *Bd*-strain infection treatments (252, 1.21% of genes, model *skin alive* in Supplementary Table S2, Supplementary Fig. S6). This was also visible in the clustering of the top 30 differentially expressed genes in skin samples (comparison: infected vs control individuals, Fig. 1), where the two *Bd* treatments cluster together. Interestingly, nine of these genes were related to immune functions, mainly interferons and the major histocompatibility complex, and were upregulated in infected individuals (Supplementary Table S6). Many genes upregulated in infected individuals were enriched for immune-related functional terms, such as interleukins, T-cells, and B-cells. The pattern was not affected when samples were split by treatment instead of infection status (Fig. 2a, Supplementary Table S6). When dividing the samples by population and conducting the same comparison between infected and control individuals, a notable finding emerged. Specifically, genes from individuals from the northern population were enriched for a higher number of terms, including immune-related ones, while the *proportion* of immune terms remained remarkably consistent (Fig. 2, Supplementary Table S7). Functional terms related to blood coagulation and wound healing were depleted in skin samples from infected individuals. Repeating the analysis using the *Xenopus tropicalis* database on g:Profiler yielded much fewer functional annotations, but a persistent pattern of similar expression between treatments and more immune related terms in the north (Supplementary Fig. S8).

### Differential gene expression in liver samples from surviving individuals

**Figure 3:**
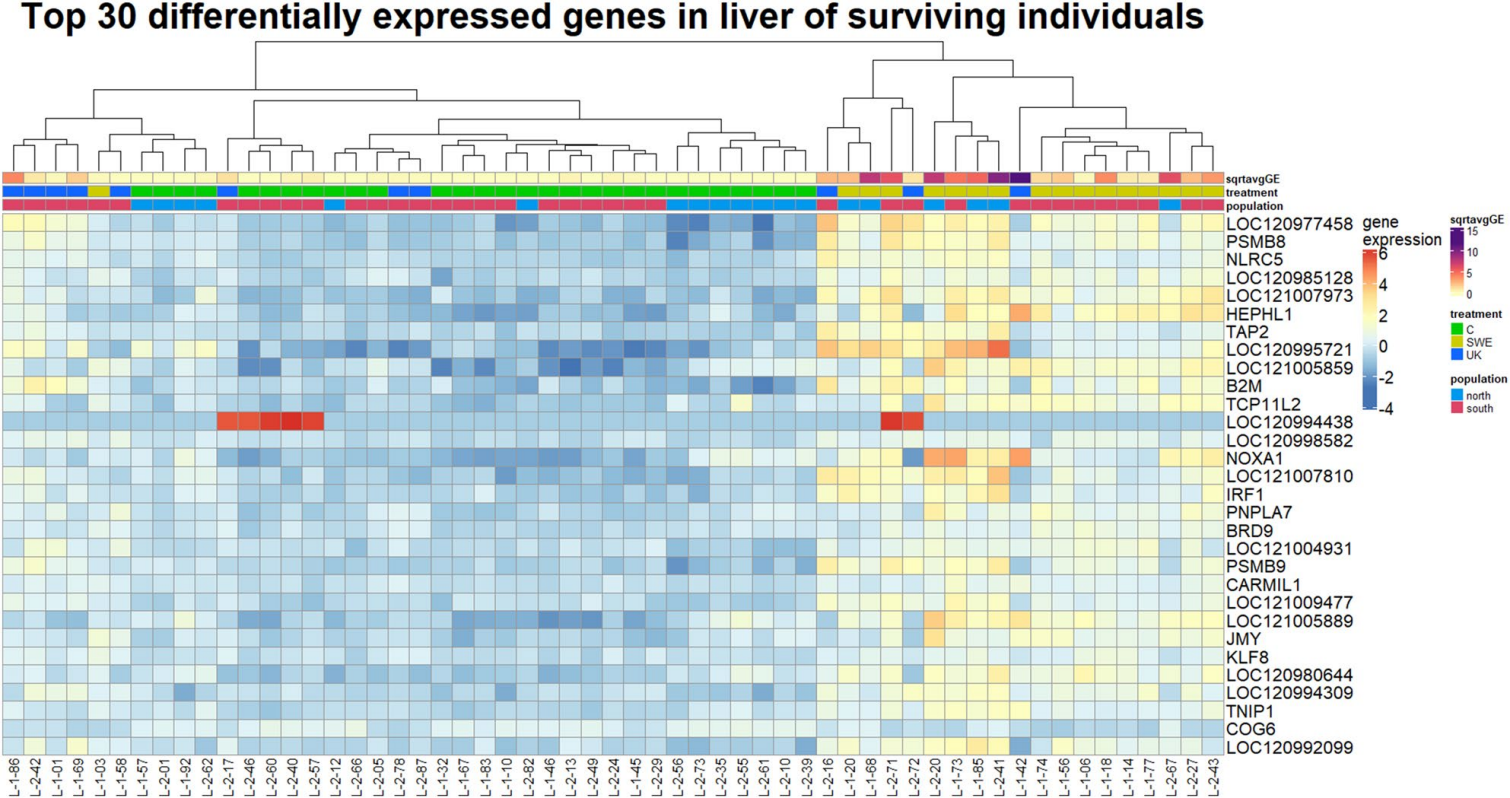
Heatmap of top 30 differentially expressed genes in liver samples of surviving individuals. Gene expression is normalized counts centered around the mean expression for each gene. The dendrogram is a hierarchical clustering based on sample similarity. sqrtavgGE: the square root of the average genomic equivalents of Bd (see Meurling et al.^37^); the minimum value (0) indicates individuals with no detectable Bd load, treatment: *C* = control, SWE and UK Bd strain, population: north or south. Heatmap generated with the R package ComplexHeatmap, version 2.14.0.

Liver samples from surviving individuals were less differentiated by infection status compared to the skin samples (6537, 33% of 19,906 genes differentially expressed, model *liver alive—inf status* Supplementary Table S2, see also Fig. 2, Supplementary Fig. S7, Supplementary Table S6). In the heatmap of the top 30 most differentially expressed genes between infected and control individuals, the two infection treatments cluster separately: individuals infected with the Swedish strain are separated from both controls and individuals infected with the UK strain (Fig. 3). It is noteworthy that individuals infected with the Swedish strain also had a larger infection load compared to those infected with the UK strain (Fig. 3). Compared to skin, the general expression between infected and control individuals yielded fewer functional terms related to immunity (61 vs 393 terms in skin, Fig. 2a, Supplementary Table S6). Separating the samples by treatment instead of infection status revealed that overexpressed genes in individuals infected with the Swedish strain were enriched for a multitude of immune related terms, though still less so than for skin samples. As was hinted by the heatmap for the top 30 genes, comparing the UK infected individuals to the controls yielded no immune related enriched terms (Fig. 2a, Supplementary Table S6). The pattern of the UK *Bd* strain eliciting no immune response reappears when analyzing only liver samples from the south (Supplementary Table S8), but cannot be examined in the North, since there are no samples from surviving individuals infected with the Swedish strain in the North. Splitting the samples by population alone and repeating the comparison between infected and control individuals, revealed that individuals from the north were enriched for a significantly higher number of terms overall, but the percentage of immune terms was often lower in the north (Supplementary Table S7). As with skin, far fewer functional terms were discovered using *X. tropicalis* as a reference in g:Profiler, compared to using *H. sapiens*, and most of them were only discovered in samples from northern toads, infected with the Swedish *Bd* strain (Supplementary Fig. S8).

### Overlap of differentially expressed genes between treatments and populations

**Figure 4:**
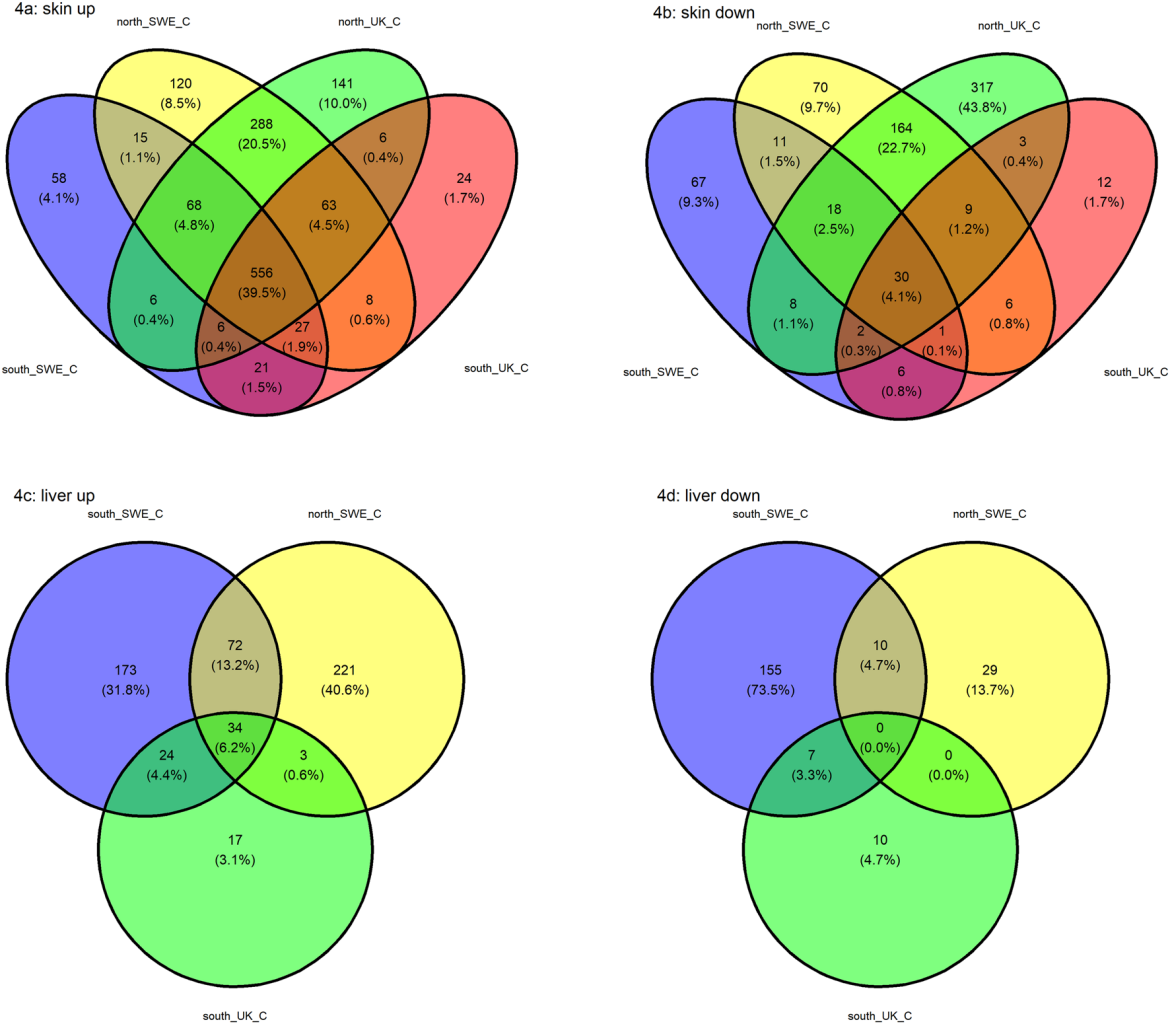
Venn diagrams for skin (**a**,**b**) and liver (**c**,**d**) samples of surviving individuals. Number and percentage of up and down- regulated genes in individuals infected with either Bd strain (UK or SWE) compared to control individuals, split by population.

In skin samples obtained from surviving individuals, a substantial proportion (39.5%) of upregulated (compared with their control counterparts) genes exhibited a shared presence across different populations and treatments (Fig. 4). The northern populations shared the second largest amount of common genes, 20.5% or 288 genes. There was negligible overlap within treatments, with only 15 genes (1.1%) shared between northern and southern individuals infected with the Swedish *Bd* strain and six genes (0.4%) for the UK strain. In the liver samples, a lower percentage of genes (34 genes or 6.2%) was common across treatments and populations while 13.2% of the genes (72 genes) were shared between individuals infected with the Swedish *Bd* strain. There were no northern individuals infected with the UK strain that survived the experiment.

Analyzing the GO terms linked to those unique genes, it was observed that skin samples from individuals infected with the Swedish strain exhibited enrichment in the "complement component C1q" term. Notably, two out of the fifteen genes were found to encode the complement C1qA and C1qC chains (Supplementary Table S9, *s_SWE_UP*). Infected individuals were overexpressing genes related to interleukin-17 in the northern population, which were not found in the rest of the skin samples. Moreover, many of the liver genes differentially expressed in individuals infected with the Swedish strain were immune-related, for example interleukin and T-cell receptors (Supplementary Table S9, *l_SWE_UP*). At the same time, only two (IL21R and LOC120977458) genes in common between all infected individuals in liver were overexpressed immune-related genes (Supplementary Table S9, *l_all_UP*).

## Discussion

We experimentally infected *Bufo bufo* metamorphs with two strains of the fungus *Batrachochytrium dendrobatidis* and investigated the expression patterns in skin and liver for individuals from populations from the south and north of Sweden. We found that expression differed by tissue and survival status. In the skin samples of the individuals that survived the experiment, expression patterns were affected by infection status and population. For liver, *Bd*-strain treatment and population strongly affected gene expression.

As *Bd* infects the skin of amphibians, we generally expect a more intense (i.e. more genes are overexpressed) and immune-targeted (i.e. many immune-related terms) response in the skin^39,40^. Early research based on microarrays was only detecting a skin response, while other amphibian tissues were seemingly unaffected by the infection^41^. Later studies, including the present one, have showed a response from other tissues, such as liver or spleen (e.g. Ref.^27,28,42^. In our experimental individuals, the liver response was less pronounced compared to the skin response, in accordance with the observation that liver generally has a broader gene expression and is not a primary immune related organ.

We found large differences in gene expression between individuals that survived until the end of the experiment and those that did not. A possible explanation for this observation is that, due to experimental constraints, skin and liver from dead individuals was not harvested at the same time after death. Some individuals were euthanized when they exhibited irreversible signs of chytridiomycosis (i.e. loss of righting reflex) and sampled at max. 2 h after euthanasia, while others were found dead on our daily checks and sampled possibly several hours after death. The lifetime of mRNA in cells is variable, so we were unable to pick up clear signals from dead individuals. It is perhaps due to this constrain that we were unable to pick up any immune-related signal from dead individuals, and only detected an enrichment of some stress-related functions and glucocorticoid levels in skin samples from dead individuals. A second possibility for the lack of any immune signal from the toadlets that did not survive, is that we are observing the immunosuppressive capability of *Bd* which has been reported in several studies^13,28,43^. Further experimental studies comparing surviving individuals with different infection loads could be valuable for identifying and measuring the presence and extent of *Bd* immunosuppression.

### Skin function and response to infection

The skin of amphibians is responsible for oxygen intake, osmoregulation, and ion exchange^9^. Aside from the many functions the skin serves in a healthy animal, it also acts as the first line of defense against pathogens. There are several immune pathways and genes active in the skin which defend against disease at this point of first contact, including the MHC and antimicrobial peptides^13,14,44^. Chytridiomycosis begins with skin hyperkeratosis and cell death^45,46^, and can quickly lead to cardiac imbalances^10,47^ and damage in osmoregulation^48^ and hepatic function^49^. Thus, a swift response is needed to counteract *Bd* infection when and where it begins.

We observed a strong skin response in infected individuals that survived until the end of the experiment compared to their control counterparts. Upregulated genes in infected individuals were enriched for a multitude of immune terms (Fig. 2) related both to the innate (e.g. cytokines, macrophages) and adaptive (e.g. B and T – cells) immune system. The skin response did not differ much between the two *Bd* strains, potentially because the response is largely generalized. However, individuals infected with the Swedish strain were enriched for a complement component, which enhances other immune factors^50^ and could in part explain the higher survival of individuals infected with that strain (Table 1, see also Meurling et al.^37^. The skin response was also similar in individuals from the south and the north of Sweden, but with more terms enriched in northern compared to southern individuals (Fig. 2), for example more interferon, interleukin and mast cell associated terms. For example, interleukin-17, which is a proinflammatory cytokine^51^, was only enriched in northern individuals and may indicate an ongoing infection in those individuals surviving until the end of the experiment.

### Gene expression in skin and association with mortality and infection load

Given that disruption of skin function caused by *Bd* infection leads to death, it is noteworthy that the northern toads experienced higher mortality than southern toads^37^ *without* major differences in skin gene transcription. This is in line with the results of previous studies, which have found little to no differential expression between skin samples of infected frog populations with different levels of susceptibility to *Bd*, while major differences were seen in other organs, such as the spleen^13^. Meurling et al.^37^ discussed the implications of different developmental strategies between the northern and southern populations on the body mass of toadlets and the development of the amphibian immune system. In summary, higher larval development rates are selected for in higher latitudes, which may trade-off with disease resistance^52^. Here, it is hinted that, indeed, very few individuals in the north have the capacity to survive infection by maintaining a strong skin immune response, potentially as a result of the trade-off between development and immunity. Further proof would require tissue sampling at frequent time intervals to capture gene expression at early infection stages.

Additionally, we discovered that, apart from immune related genes, chytrid infection influenced genes related to blood coagulation. These genes were downregulated in infected individuals compared to controls, and the GO terms indicate that these genes are either related to a *negative* regulation or otherwise have no indication of the direction of the effect. This results in a net positive reaction for infected individuals, which appear to be actively trying to regulate the skin lesions created by *Bd*. This has also been observed in an infection experiment with the putatively *Bd* resistant species, *Bufo marinus*, which overexpressed genes related to skin structure maintenance^27^. Further research might benefit from immunohistochemistry analysis of the skin. Tissue sections could provide a more complete picture of the structure of infected tissue and help determine if there are any population-related differences in this tissue which are not captured by RNAseq.

### *Bd* strain effects on liver gene expression and disparity with treatment effects on skin

The response to *Bd* infection shows a different picture in liver. While *Bd* strain was not important in determining gene expression in the skin, in the liver it played a major role. We found upregulation of immune genes in liver samples of individuals infected with the Swedish strain of *Bd*, while this was not the case in individuals infected with the UK strain. Previous studies have uncovered such differences between strains in other response variables. Berger, Marantelli et al.^53^ observed differences in the survival time of *Litorea caerulea* metamorphs experimentally infected with three different *Bd* strains. Gabor et al.^54^ used two different lineages of *Bd* (*Bd*-GPL and a less virulent lineage, *Bd*CAPE) and observed that *Alytes obstetricans* tadpoles released higher amounts of corticosteroids, associated with the amphibian homeostasis and immune response, when infected with the hypervirulent *Bd*-GPL.

In our study, we found differences in expression pattern between strains supporting previous studies by Meurling et al.^37^ where differences in pathogenicity between the strains were uncovered and infection load was higher in toads infected with the Swedish strain. The UK strain has been passaged many more times compared to the Swedish isolate^7^. Passaging is known to decrease the pathogenicity of *Bd*^55^, so it is possible to imagine that reduced pathogenicity in which the first line of response (skin) is activated, but no noticeable liver response is elicited. However, the decreased virulence hypothesis is refuted when considering the fact that the UK strain actually is more lethal than the Swedish one. Another explanation is that the UK strain, having undergone many more cycles of division, has accumulated mutations that allow it to escape detection from the liver response mechanisms. It is important to remember that spleen is the primary lymphoid organ in the immune system^14^. Liver, which is a secondary lymphoid organ, was chosen in this study because of the difficulties in isolating spleen in the very small *B. bufo* metamorphs.

Our results suggest that the difference between *Bd* strains on liver samples was not driven by population differences. As no infected individuals with the UK strain survived to the end of the experiment, it is impossible to compare surviving individuals infected with different strains separately in the north. Nevertheless, in the south we observe the same pattern of expression in individuals infected with the Swedish strain being different to both UK-infected individuals and controls (Fig. 2).

The genes overexpressed in SWE-infected individuals compared to controls are fewer compared to skin (Supplementary Table S2), and enriched for fewer immune related terms (Supplementary Table S6). This is probably due to liver having a much broader expression pattern, being involved in, not only lymphopoiesis^14^, but also metabolism, bile productions, glycogen storage etc.^38^.

### Population differences in liver gene expression

Alongside the pattern of southern individuals showing a clear strain effect in the liver, we detected a significant difference in expression between southern and northern populations overall, with northern liver samples being enriched for many more terms. This reflects the enrichment pattern as observed in the skin, and is even more pronounced in liver. This is interesting given the disparity in mortality between the two populations. As the toads in this experiment were raised from eggs in a laboratory setting, ruling out the possibility of environmental factors as the cause of different responses across treatments and populations, it is possible that these differences reflect genetic differences between the northern and southern population, shaped by demographic processes.

Different genetic responses to stressors can lead to varying disease phenotypes and ultimately translate into different survival outcomes. Previous studies have suggested that while environmental variables shape the intensity of *Bd* infection, genetics may ultimately be the strongest factor determining survival^13,56^. In the present study, deaths among infected northern toads accounted for 87% of deaths overall, suggesting a genetic disadvantage compared to the southern population. A similar study by Savage et al.^13^ found that infected *Rana yavapaiensis* that exhibit a sustained immune reaction to *Bd* have worse survival outcomes than individuals with a more mild response. It is still largely unknown why this is the case, but it is speculated that inflammatory genes could trigger an autoimmune response, similar to a cytokine storm^13,57^. Additional histological investigation would be necessary to confirm whether this occurred in the northern population toads included in the present study. It is also possible that northern toads are naïve to *Bd* infection, though only few populations have been scanned for infection in the north so far^58^. Earlier studies found that frogs from populations that have been exposed to *Bd* for a long time, can mount an earlier and more robust response and thus be able to survive under chytridiomycosis^42,59^. A late response to chytridiomycosis has also been found to be non-protective^24,26^. This is in line with our observation that gene expression differences are bigger between infected and control surviving individuals in the north (Fig. 2), while survival is lower.

### Examination of the top 30 differentially expressed genes in skin and liver

Upon closer analysis of the genes displayed in Figs. 1 and 4, we found that several genes were associated with immune response (Supplementary Table S10), including genes associated with the MHC. Genetic variation within the MHC peptide binding region can provide varying degrees of resistance to chytridiomycosis within populations and species^60^. In populations with persistent outbreaks, *Bd*-resistant MHC genes can provide a selective advantage and result in directional selection over time^61,62^. TAP1, TAP2, PSMB8 and PSMB9 constitute components of the MHC pathway and adaptive immune system. The upregulation of these genes in infected *B. bufo* skin and liver samples suggests this pathway is highly active when *Bd* is present. TAP1 and TAP2 are ABC transport proteins which form a complex that translocates antigens to the endoplasmic reticulum; from there, antigens are transferred to MHC molecules that later bring them to the cell surface for degradation by T-cells^63^. Additionally, PSMB8 and PSMB9 are both subunits of a proteasome encoded by MHC genes which preprocesses antigens^64^. Previous studies have found MHC-related genes to be upregulated in the skin of amphibian species which are less resistant to *Bd* infection^13,25^. In the case of *B. bufo,* upregulation of MHC genes confers protection to *Bd*, as this is found in infected but surviving individuals. Also, solute carrier gene SLC11A1 is within this group, having associations with inflammatory disease and immunity^65,66^. While it may seem surprising that there is not greater overlap in the immune response between the two tissues, organ-specific immune responses to infectious disease are common in vertebrates^67^.

## Conclusions

The degree of biodiversity loss caused by wildlife disease is staggering and worthy of comprehensive and rigorous investigation. Given its prevalence and transmissibility, *Bd* will continue to pose a threat to a wide array of amphibian species for many years to come. Understanding the factors which exacerbate chytridiomycosis pathogenicity can help inform mitigation strategies and conservation.

We were able to gain insight into the functions of many differentially expressed genes in the common toad and better understand how some tissue types respond to *Bd* infection. The pronounced gene expression differences we discovered between surviving and dead individuals for both tissues echo previous findings which have demonstrated that *Bd* infection can lead to severe misregulation of skin and liver metabolites^68^, while we also uncovered *Bd* strain and host population effects on gene expression.

Genetic characteristics that confer disease-resistance should be studied further. We emphasize the need to examine differential survival and gene expression in response to infection at both the species and population level to gain valuable insight into host–pathogen dynamics and the underlying physiology which determines survival. This study provides valuable information regarding the response of amphibians to *Bd* infection and serves as a basis for further research that will include the effects of factors such as rearing temperature and skin microbiome.

## Materials and methods

### Husbandry – infection experiment

The procedures for animal husbandry and the infection experiment are described in detail in Meurling et al.^37^. Briefly, we collected *Bufo bufo* eggs from four ponds, two in the south and two in the north of Sweden (Supplementary Table S11). Permits for egg collection were obtained from the county administrative boards in Skåne and Norrbotten. The eggs were kept in climate-controlled rooms at 19 °C, and the hatched tadpoles were fed spinach and fish flakes *ad libidum*. After metamorphosis, we kept toadlets at the same temperature in individual boxes, and fed them fruit flies and crickets. We infected metamorphs with a high dose (60,000 zoospores) of either the Swedish or UK *Bd*-GPL strain or sham infected them with *Bd* growth medium, and monitored them for 30 days for signs of chytridiomycosis. At the experimental endpoint (death/disease, or after 30 days had passed), individuals were euthanised with an overdose of MS222. Skin and liver were harvested from every individual and the tissues were stored in RNAlater until RNA extraction. All applicable institutional and/or national guidelines for the care and use of animals were followed. The Uppsala ethical committee for animal experiments provided the permit for the experiments (permit number C28/15). No additional animal experiments were conducted for this paper, we only used tissue samples from the experiment described above. Our study is reported in accordance with ARRIVE guidelines^69^.

### RNA extraction, library preparation and sequencing

We used the RNeasy Mini kit (Qiagen) according to the manufacturer's instructions, for RNA extraction from 181 skin and liver samples collected from the experimental individuals. The quality of the extracted RNA was assessed on a Bioanalyzer instrument, with the RNA 6000 Nano Kit (Agilent). For library preparation, we used the TruSeq Stranded mRNA library prep kit (Illumina) on samples with RIN > 7.5, following manufacturer’s instructions, with the addition of an extra Ampure XP (Beckman Coulter) magnetic bead wash at the end of the protocol to remove primer dimers (see Appendix 1). We assessed library quality on the Bioanalyzer, using the DNA 1000 Kit (Agilent) and measured final DNA concentration on Qubit. Finally, we pooled libraries in equimolar amounts and performed paired-end 150 bp sequencing on two lanes of a DenovoSeq S4 flow cell with v1 sequencing chemistry at SciLifeLab in Sweden.

### Bioinformatics

We assessed the quality of the obtained reads using FastQC^70^ and MultiQC^71^, prior to and after using Trimmomatic^72^ to trim adaptor regions from sequences and discard low quality and very short (< 50 bp) reads. We indexed the *B. bufo* genome assembly and annotation^73^ using STAR^74^ and afterwards mapped all sample reads that passed the Trimmomatic quality checks to it. To create the data matrix to quantify gene expression for every sample we used RSEM (Li & Dewey^75^) on the aligned reads. Bioinformatic software and parameters, as well as R package versions are provided in Supplementary Table S12.

### Differential expression analysis in R

The data matrix and metadata for the samples were loaded into R Studio (version 2023.03.2 + 454, R version 4.2.2)^76^ for differential expression analysis. We rounded read counts to the nearest integer value and filtered out samples with less than 500,000 reads in total. The R package *DESeq2*^77^ was used for the analysis. For the models explored, we built a DESeqDataSET object, with either a null model design, or a design with one or more explanatory variables (among region, tissue, death, *Bd* treatment, and infection status). We also pre-filtered low count genes (keeping genes with > 10 reads across all samples) and performed variance stabilizing transformations. Preliminary results were visualized using principal component analysis (PCA) with the function *plotPCA* from the *DESeq2* package. After preliminary analysis, we split the dataset per tissue, and conducted separate analyses for liver and skin (Supplementary Fig. S2). For some of the analyses, we combined the Swedish and UK-strain infected groups into a single infected group for comparison against controls.

For the differential expression analysis for liver and skin samples we used the function *DESeq* on each DESeqDataSET object and generated result tables with log2 fold changes and (adjusted) p values using the function *results*. The model contrasts were specified to obtain results for differential expression between different sample combinations (e.g. north vs south, or dead vs surviving individuals). As a diagnostic, we plotted dispersion estimates for each model using the function *plotDispEsts*. Genes significantly up- or down- regulated between different combinations of conditions (p ≤ 0.05, |log2fold change|≥ 2) were recorded. The main differences tested were (a) between surviving and dead individuals, for each tissue, as well as within each population and treatment and (b) between control and infected individuals among the ones that survived until the end of the experiment. We visualized some of these differences using the function *EnhancedVolcano* from the package of the same name. Additionally, we created Venn diagrams, using the function *ggvenn* from the package *ggvenn*^78^ in order to examine the overlap of differentially expressed genes between surviving, control and infected individuals in each population and treatment combination. We also created heatmaps of the top 30 differentially expressed genes between infected and control living individuals for each tissue using the package *ComplexHeatmap*^79^. R package versions, as well as software versions and parameters are provided in Supplementary Table S12.

### Functional enrichment analysis

We performed functional enrichment analysis for the differentially expressed genes for different combinations of predictors using the g:GOST function of the online tool g:Profiler (v e108_eg55_p17_9f356ae, https://biit.cs.ut.ee/gprofiler/gost) with the *g:SCS* multiple testing correction method, and applying a significance threshold of 0.05^80^. We searched in the Gene Ontology (GO) and biological pathways databases and used two databases as reference: *Homo sapiens*, which was the most complete dataset, as well as *Xenopus tropicalis*, as the amphibian database.

### Supplementary Information

Supplementary Information.

Supplementary Tables.

## Data Availability

These raw data associated with this article have been submitted to the NCBI Sequence Read Archive and can be found under accession number PRJNA1019526, at http://www.ncbi.nlm.nih.gov/bioproject/1019526 (to be released).
