# Supplementary material for: Batrachochytrium dendrobatidis strain affects transcriptomic response in liver but not skin in latitudinal populations of the common toad (Bufo bufo)
Source: Sci Rep. 2024 Jan 30;14:2495. doi: 10.1038/s41598-024-52975-8 (PMC10828426; doi:10.1038/s41598-024-52975-8)
Supplement: Supplementary file 1 — Supplementary Information. [file 41598_2024_52975_MOESM1_ESM.pdf]

Batrachochytrium dendrobatidis strain affects transcriptomic response in liver but not skin in latitudinal populations of the common toad (_Bufo bufo_)

Niki Chondrelli, Emily Kuehn, Sara Meurling, Maria Cortázar-Chinarro, Anssi Laurila, Jacob Höglund

Table of contents

Table of contents

Supplementary figures 3
Figure S1. 3
Figure S2. 4
Figure S3. 5
Figure S4. 6
Figure S5. 7
Figure S6. 8
Figure S7. 9
Appendix 1 11

Supplementary figures

Figure S1. PCA of the top 500 differentially expressed genes in the experiment, colored by tissue. Plot drawn after removal of samples with low gene counts. Notice the sample labeled as liver, which clusters with the skin samples. That sample was removed from further analysis, as it was probably mislabeled. Model design for the PCA: ~ tissue + population * treatment + death_factor.

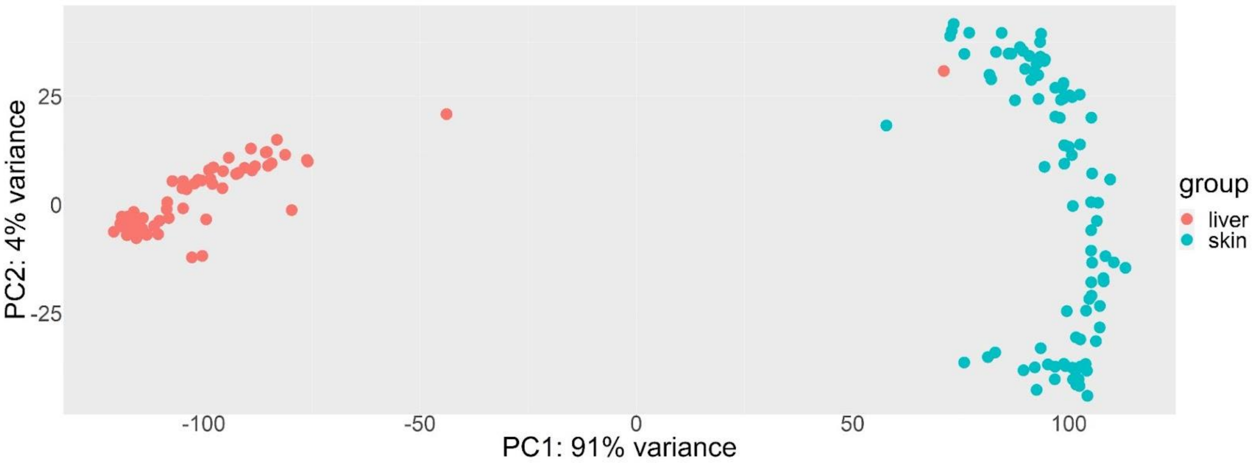

Figure S2. PCA of the top 500 differentially expressed genes in the experiment, coloured by tissue, plotted after the removal of a potentially mislabeled liver sample clustering with the skin samples (see figure S2). Model design: ~ tissue + population * treatment + death_factor.

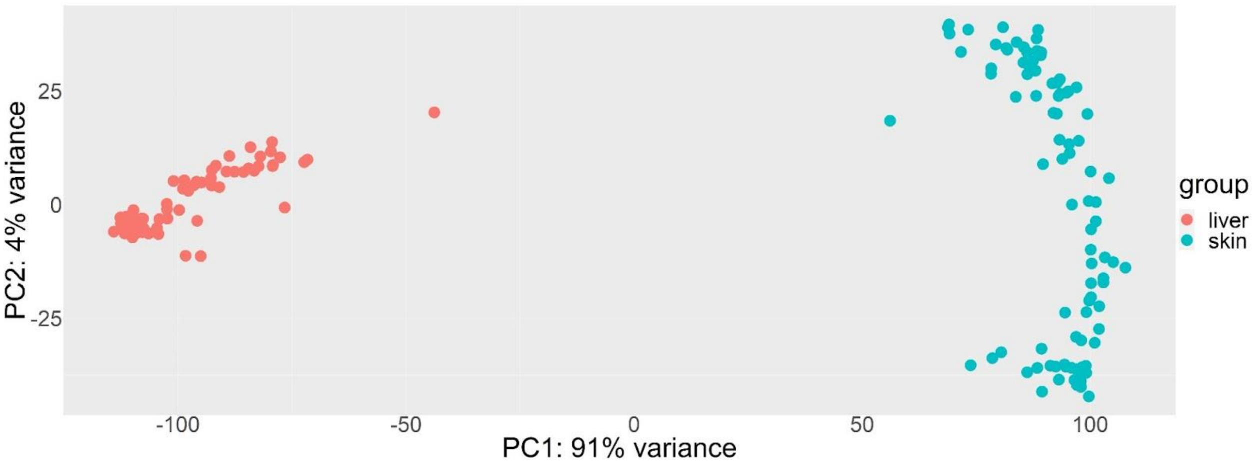

Figure S3. Enhanced volcano plot for all the differentially expressed genes in the experiment, with “tissue” as the contrast (reference level “skin), and model design: expression ~ tissue + population * treatment + death_factor.

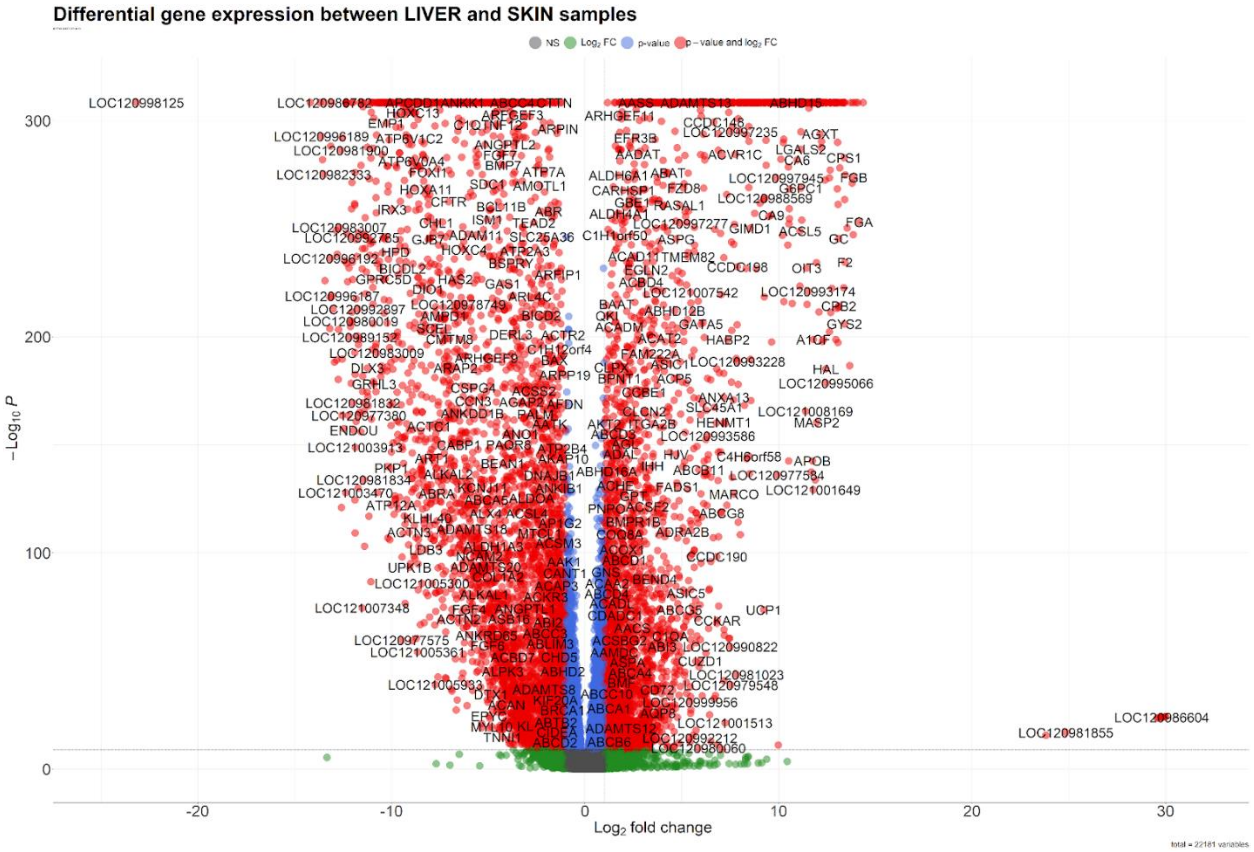

Figure S4. PCA of the top 500 differentially expressed genes in the skin, colored by treatment (control, SWE or UK *Bd* strain), death_factor (a factor variable with two levels, alive or dead by the end of the experiment) and population (north and south) plotted after the removal of a potentially mislabeled liver sample clustering with the skin samples. Model design: population * treatment + death_factor. Ellipses are drawn for all groups, except "UK:alive:north" which consists of only one sample.

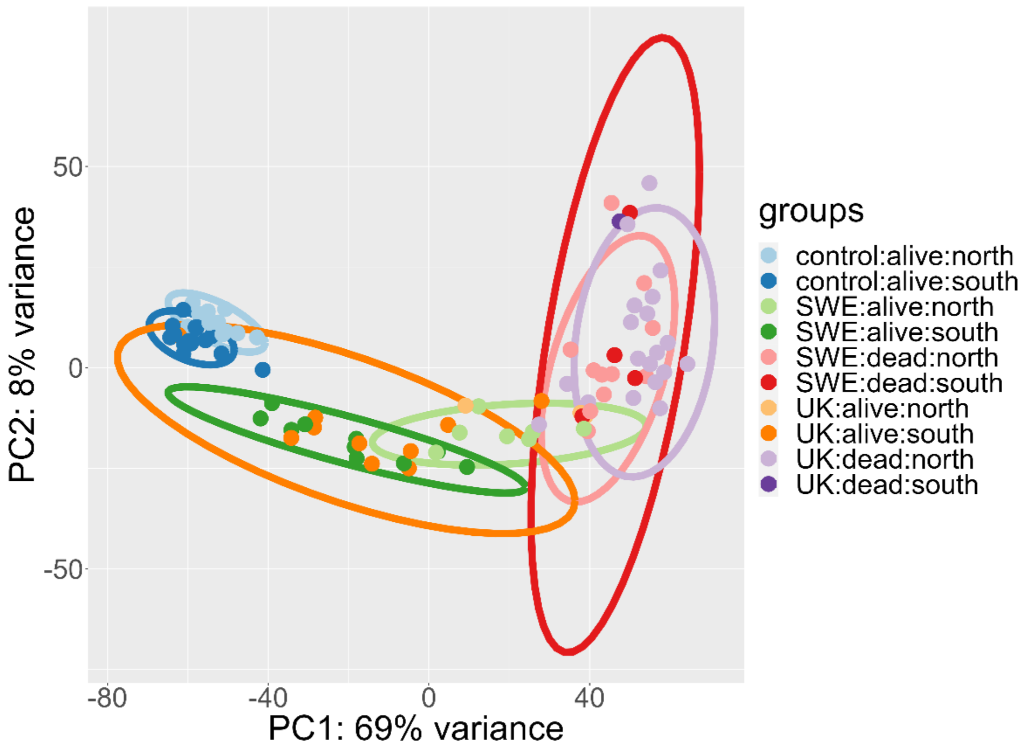

Figure S5. PCA of the top 500 differentially expressed genes in the liver, colored by treatment (control, SWE or UK *Bd* strain), death_factor (a factor variable with two levels, alive or dead by the end of the experiment) and population (north and south) plotted after the removal of a potentially mislabeled liver sample clustering with the skin samples. Model design: population * treatment + death_factor. Ellipses are drawn for all groups, except "SWE:dead:south" which consists of only two samples. There are no samples from southern individuals infected with the UK strain that died, or from northern individuals infected with the UK strain that survived.

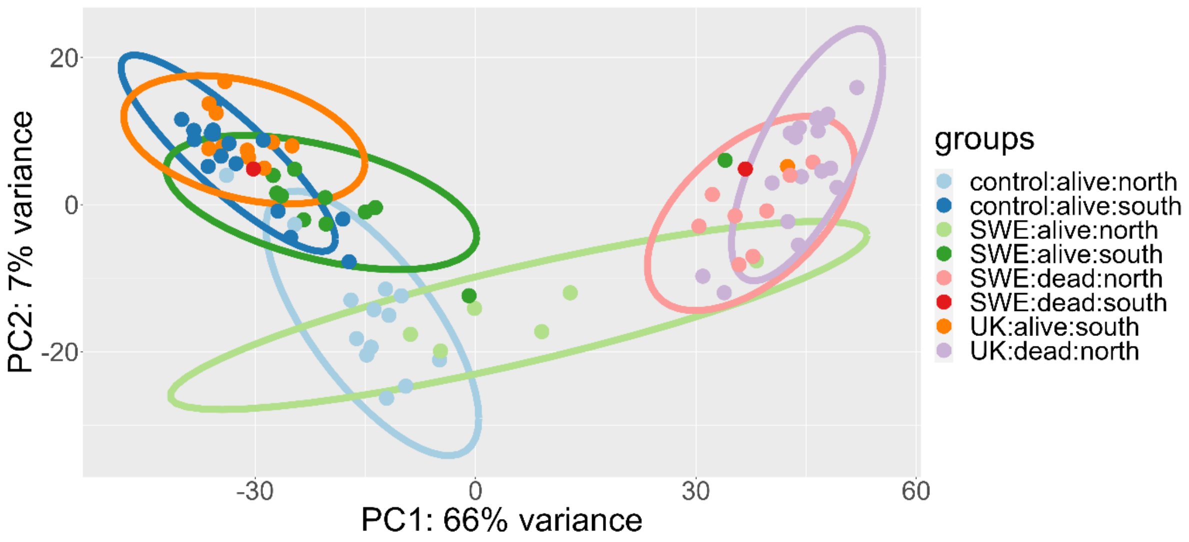

Figure S6. PCA for top 500 differentially expressed genes in skin samples from alive individuals, colored by population (North and South) and treatment (control, UK or swedish *Bd* strain). Model design: population * treatment

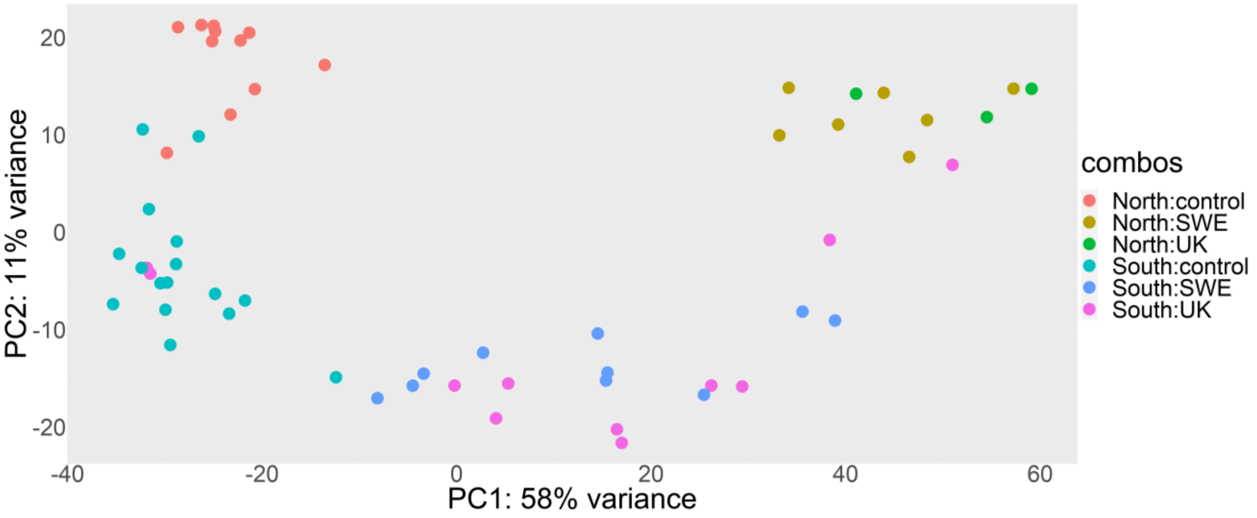

Figure S7. PCA for top 500 differentially expressed genes in liver samples from alive individuals, colored by population (North and South) and treatment (control, UK or swedish *Bd* strain). Note that there are no surviving individuals in the north infected with the UK *Bd* strain. Model design: population * treatment

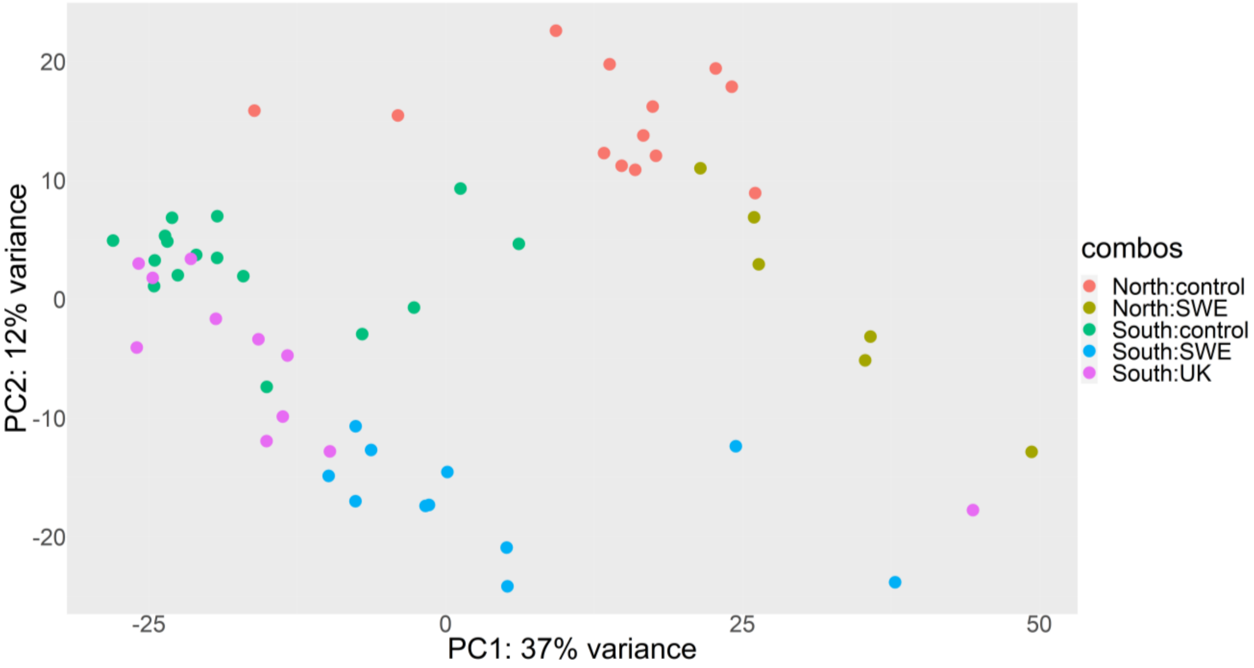

Figure S8. Number (top) and percentage (bottom) of immune related search terms in comparisons between infected and control surviving individuals using the *Xenopus tropicalis* database in g:Profiler. The data is split by tissue (skin – liver), population (north, south or both) and treatment (all inf.: all infected individuals, SWE: individuals infected with the Swedish Bd strain, UK: individuals infected with the UK Bd strain. All comparisons are with control individuals of the same category. Percentage (bottom) refers to the percentage of immune related terms over all the gene ontology terms related to biological processes and enriched in each comparison).

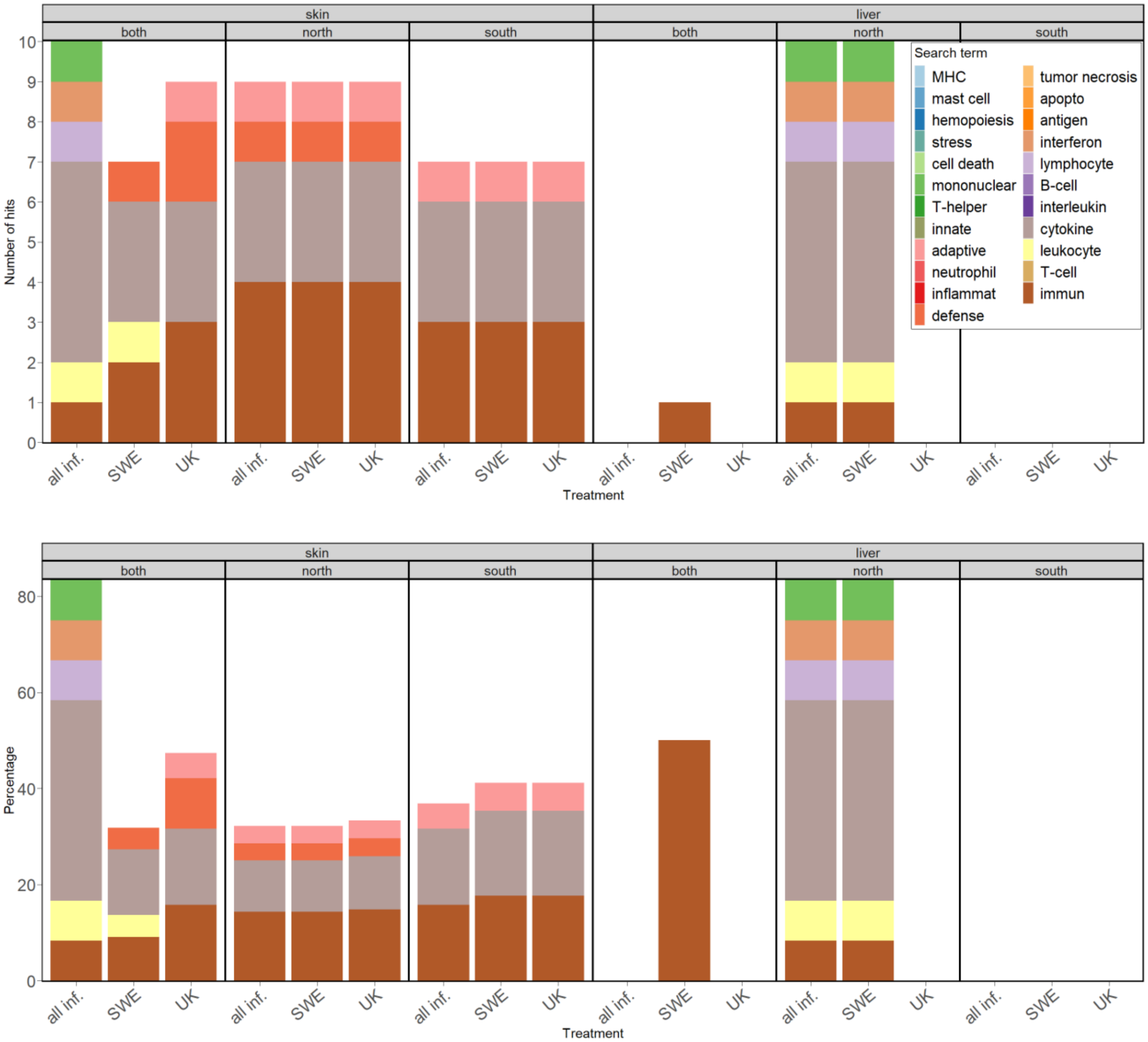

Appendix 1

The extra wash was carried out as follows: 24uL of Ampure XP beads were added to the 30uL of DNA in resuspension buffer (RSB), mixed by pipetting, and incubated at room temperature for 15 minutes. Supernatant was discarded and the pellet was washed twice with 200uL 80% ethanol before being left to air dry for 15 minutes. 32.5uL RSB was added and mixed by pipetting. Samples were returned to the magnetic stand and supernatant was collected.
